# A qualitative study on patient-centered care and perceptions of nurses regarding primary healthcare facilities in Nigeria

**DOI:** 10.1186/s12962-022-00375-y

**Published:** 2022-08-13

**Authors:** Monsurat Adepeju Lateef, Euphemia Mbali Mhlongo

**Affiliations:** 1 Department of Nursing, College of Health Sciences, University of KwaZulu-Natal, Howard College, Durban, South Africa; 2 Department of Nursing, College of Health Sciences, University of KwaZulu-Natal, Howard College, Durban, South Africa

**Keywords:** Patient-centered care, Perception, Nurses, Primary Health Care, Nursing care, And Healthcare services

## Abstract

**Background:**

Patient-centered care (PCC) approach has continued to gain recognition globally as the key to providing quality healthcare. However, this concept is not fully integrated into the management of primary health care (PHC) in existing nursing practice due to numerous challenges. Among these challenges is the perception of nursing on PCC in the Primary Health Care system. This study seeks to present the results of qualitative research performed at various selected PHC centres on nurses’ perceptions in PCC practice. This study aim was to explore the perception of nurses on PCC.

**Methods:**

A qualitative action research approach was adopted. The study involved 30 local government PHC centres located in Osun State Southwest of the federal republic of Nigeria. Data was collected through a semi-structured interview guide questions. Thereafter, data analysis was performed using thematic analysis and NVivo 12 software to generate themes, subthemes, and codes.

**Results:**

PCC perceptions of nurses that was revealed in our findings were categorised into positive and negative themes. The negative themes include: poor approach by the nurses and lack of enforcement agency. The positive themes that emerged include: outcome driven healthcare, valued care provider, communication to sharpen care and driven healthcare service.

**Conclusion:**

There is need for continuous training, and upgrading of nurses in line with global recommended standards of providing quality healthcare service delivery to the people. Therefore, the federal and state governments and local government council through the Nursing and Midwifery Council body should regulate, supervise, monitor and enforce the use and implementation of PCC in the PHC healthcare system.

## Background

Patient-centered care (PCC) was developed in the 1950s by Carl R. Roger, an American humanist psychologist in what he referred to as client-centered therapy [1–3]. Today, PCC is widely recognized and acknowledged as a core foundation in nursing practice. This has led to positive outcomes such as reduction in malpractice complaints, improved patients’ satisfaction, improved consultation time, patient’s emotional state, and medication adherence [4, 5]. Giving healthcare that is geared towards PCC is needed for patients better, positive health outcome and satisfaction of care [6]. Newell and Jordan [7] reported that PCC is an essential and fundamental principles in promoting quality healthcare [7]. This is in the understanding that patients are fundamentally human, leading to the promotion of the concept of PCC which is individualised treatment, but not without complexities [8].

Historically, the concept of PCC has been explored through theoretical proposals and practical approach since the earliest Geneva conference that followed a process that allowed the acquisition of experience concurrently [9]. In essence, the experience is a crucial aspect of the identity and existence of any institution that connotes the value of delineating institutional journeys, evaluating and recognizing PCC development.

A systematic review on PCC reported that PCC concept is a comprehensive approach to healthcare service delivery. It has a direct link to patient’s high quality care [10]. This link gives room for sharing of power and responsibilities between the patient and the caregiver when patient is perceived and treated as a person with biopsychosocial needs [10].

This innovative concept in policy and practice moving to a conceptual optimal routine clinical practice is pivotal [11, 12]. In view of the benefits of PCC, this study is important because currently, healthcare service delivery in Nigeria PHC is not geared toward the needs of the patients. This study will contribute to improving PHC healthcare system, nursing practice and patient satisfaction.

In developed countries such as the United Kingdom, United States of America, Australia, and Germany, PCC has been in paper through policies leading to this statement, “*liberating the NHS: No decision about me without me*” [13, 14]. Even though the trend of this concept is clear in research and theoretical conceptualization. It is, however, fuzzy, elusive, and even poorly implemented in medical and nursing practice [15–17]. Since its inception, several studies on PCC such as [4, 18–20] have described the various dimensions and models of PCC to show its importance in the healthcare system. In addition, several research findings on the challenges experienced in realizing and practicing PCC have been presented in [21], among others. The results of these findings also reveal that PCC has been taken for granted by healthcare providers and other stakeholders in primary health care.

In order to achieve sustainable healthcare coverage, implementation of the PCC concept is a necessity for all in Nigeria, where it has been accepted for incorporation into the existing health system. This means that it is feasible at the grassroots level of the health system provided that the stakeholders work in collaboration with the nurses and other healthcare givers in its implementation and practice. Thus, the aim of this study was to explore the perception of nurses on PCC in public health care.

PCC is a multidimensional approach to healthcare service delivery. It is underpinned with the philosophy of individuality or personhood with the goal of delivering quality healthcare to every person that receives care [22]. Universal health care according to WHO is that every citizen whether poor or rich should have access to quality healthcare service [23], in order to provide basic human right. However, this is failing still in many countries at the organisational level [24, 25].

Although, PCC is an individualised care [22], it allows healthcare providers to improve patient healthcare approaches [26]. However, it is a crucial matter because it involves the patient trust and the nurse who is the healthcare provider in the treatment. To gain patient trust in nursing practice requires more than leadership and competency [27]. The discipline of nursing is resounding in the health care for effective and efficient changes in the delivery of healthcare services [28]. According to the institute of Medicine the twenty-first century healthcare quality improvement points to PCC concept as the number one objective [29].

Today, nurses are task oriented instead of patient-centeredness due to many hurdles [30, 31], such as healthcare providers communication, structural factor like hospital size, work overload, and organisational factor [32, 33]. There is one resounding response to these problems for centuries in the literature of nursing that is PCC concept [34, 35].

In professional nursing circles, PCC is perceived to be an awareness of the importance of patient healthcare culture, family and friend’s involvement, incorporation of values of love and respect, and communication in all facets of patient’s care leading to accountability to the patient [36, 37]. There has been an increased need for PCC globally since its identification by the Institute of medicine of United States of America National Academies of Science as the leading contributor in the provision of quality PHC [38]. PCC is aimed at understanding the illness experiences from the patient’s viewpoint. The International Alliance of Patients Organization (IAPO) declared that PCC as a service is based on placing the patient at the centre and around the patient’s needs [39].

The role of nurses in delivering PCC in public hospitals and other healthcare facilities has become an imperative study for researchers since it is necessary to evaluate the knowledge and understanding of the nurses. In the findings by Terry in [40], nurses were found to be enthusiastic about sharing their perceptions with regards to PCC and what they perceived it to encompass. It is interesting to note that the perception of humane treatment was evident from the nurses. The components of human emotional aspects such as psychological, social, spiritual, and emotional aspects that greatly influence an individual’s health was presented [40]. For instance, the awareness of the patient’s cultural background and how to integrate the culture of a patient into the management of a patient and treatment plan were seen to be associated with PCC [40].

Therefore, in order to effectively render PCC, the nurses have to demonstrate cultural awareness [41, 42]. Culture is known to be multifaceted and dynamic, making it an important subject for the health practitioner’s especially nurses, to understand for quality and effective healthcare service in the PHC [42, 43]. To improve healthcare outcomes in PCC model, there has to be a demonstrated aptitude for cultural competence [44]. However, there are many challenges and barriers, but none is more influential than institutional and cultural-based on individual ethnocentrism [45]. This is responsible for the current socialization of healthcare professionals leading to the perpetuation of negative attitudes, stereotype behaviour towards the vulnerable and culturally diverse populations [46].

The involvement of the family in crucial patient’s health decisions is a vital component of PCC and has been evident in research findings presented in [47]. The similarities and differences have been identified in order to define and describe the level of family involvement in delivering PCC. Studies reported that out of nine different models and frameworks in which PCC was defined, family and friend’s involvement was found to be 60% [48, 49]. The values of love and respect for patients were seen to be the other aspects of PCC. This is seen as incorporating a holistic PCC when interacting with patients and family members. Dowling in Ref. [50], reports that nurses perceive love in nursing as going beyond the traditional duty of patient care and the willingness and commitment to the good of a patient before themselves. In Kvale and Bondevik study [51], it was found that treatment with love and respect among patients included aspects of listening and trusting in a patient. This makes the patients feel valued besides having a sense of control of their own healthcare process, thus helping in their recovery efforts. The display of these values of love, respect, and dignity was seen as integral to PCC and was perceived so by the nurses.

One way of expressing perception is through verbal and non-verbal communication. Nurses should be aware of the different methods of communication that should be adopted when handling patients. According to Levinson, Lesser, and Epstein [52], it is reported that communication skills form a fundamental component of PCC. Evidence has demonstrated the impact of PCC in healthcare and these include patient satisfaction, adherence to recommended treatment, and management of chronic diseases [53, 54]. However, PCC lacks clear measurement tools to clearly show its effectiveness as presented in Ref. [55]. Thus, the objective of this study is to explored the perceptions of nurses in providing PCC.

### Research question

What are the perceptions of nurses to provide PCC in the PHC facilities?

## Methods

### Study design

The study adopted a qualitative action research approach with a purposive sampling technique. Using a qualitative method for this study gives unique depth understanding views and perceptions of the participants. The participants were able to share their thoughts, feelings and individual view regarding the phenomena of interest which could have been difficult to express and gain from a closed ended question. The purposive sampling was employed due to the nature of research design and who could particulate in in-depth interviews of the study and to gather qualitative responses, which leads to better insights and more precise research results in order to achieve the aims and objectives of the study. Individual interviews from nurses in PHC centres were analysed using thematic analysis method. Individual interviews were conducted with a semi-structured interview guide. An action according to McNiff and Whitehead [56] indicates it as a deliberate openness to new processes and experiences, as such demands that the action of educational research is education itself [56]. Engaging the nurses in the community in individual interviews captured the nurses’ experiences on PCC utilisation. The way forward is motivating them to explore being in control of their discipline in the context of working with the understanding of PCC and its values in the PHC centres. The principle of deliberate openness to new knowledge and skills was added to the nurses during the field work.

The nature of action research method comes in phases which consisted of the following: plan, action, observe and reflect [57]. Plan phase was done through literature search to identify gaps in knowledge. Action phase was collecting and compiling evidence through data collection to help us understand “What” nurses practice, “How” they practice and “Why”. The Observation phase was the analysis phase of the study while reflection phase involved interpreting and explaining the observations with the support from data and existing literature.

The sample comprised of 35 nurses composed of 28 females and seven male nurses. The qualitative study data sample size was based on data saturation. This is the process during interviews that the same opinions from the participants starts to comes up again and again, until it reaches a point of no new ideas or information being observed [58, 59]. The saturation level was reached with the total of 35 participants. Then, data analysis was fully commenced.

The study was conducted in 13 local government areas (LGAs) with 30 PHC centres, both rural and urban located in Osun State, southwest. In the federal republic of Nigeria. Majority of these PHC centres were located in the rural communities of which transportations and road were a big challenge. The Federal Republic of Nigeria is divided into six geopolitical zones, namely: North East, North West, North Central, South East, Southwest, and South-South zone. South West zone comprised of the following 6 States: Lagos State; Oyo State; Ogun State; Osun State; Ekiti State, and Ondo State [60].

### Data collection and data analysis

A semi-structured individual interviews guide questions were used to obtained data from the participants in terms of their perceptions of PCC practice in the PHC. The individual interviews were taped recorded and later transcribed verbatim. Process, information about participants ‘demographic were obtained during data collection however, no details of the participants to have been able to identify individual was left on the transcripts.

Qualitative data analysis for this study employed a thematic analysis method following the six steps, as described by Braun and Clarke [61]. These steps were adopted to help develop a thorough, reflective and deliberate description of the themes and the sub-themes in the study [61, 62]. Version 12 NVivo software was also used to organise and manage the data. According to Braun and Clark [62] thematic analysis enables the researcher to listen, reflect, clarify and intuit during the analysis sessions. These six steps are: Familiarising with the data obtained; Generating initial codes; Searching for themes; Reviewing Themes; Defining and refining themes; and producing the report which guided this study.

### The rigor of the study

The rigor of the study explains the validity, reliability and the degree to which the researcher could influence the readers that the study is worthy of the data [63, 64]. Trustworthiness as proposed by Guba is employed in this study, according to Shenton. These four criteria: credibility, dependability, conformability, and transferability are considered in this study [65].

*Credibility*: To ensure the credibility of this study, an individual interview was adopted to obtain information from the participants. The individuals’ interviews lasted between 15 to 30 min per participant. All interviews were taped recorded. *Confirmability*: The Confirmability standard was ensured through the audio-recording and verbatim transcription of interview sections. *Dependability:* The comprehensive description of the technique for data collection, and data analysis explains the data dependable, stability and consistency of data obtained. *Transferability*: The transferability in this study was ensured by providing adequate data description and socio-demographic characteristics of the participants. This could enable the public to evaluate the applicability of the data to other contexts [64].

## Results

Both negative and positive themes and sub-themes on PCC, as described by the nurses emerged from this study. Participants’ perceptions were categorised as described in Tables 1 and 2, and Table 3 presents demographic of the participants. The instrument used for the data collection is presented in the appendix.

**Table 1:** The themes, sub-themes, and codes, with meaning based on negative perceptions

Themes	Sub-themes	Codes	Meaning
Poor approach by the nurses	Sentimentalism of the nurses	*…Patient-centered care consumes time, for me to treat [a]patient holistically it consumes time…*	Participants reported poor approach towards accessing healthcare system which is associated with nurses’ attitude toward the patients due to number of factors such as stress, workload, personal opinion and misconception
	Uncaring approach	*We look at the time for our patient…we don’t have enough time to listen to them, for the patients to say what they want to say. So aspect of the nursing care which is the counselling that this patient really need is not there any more*	
Lack of enforcement agency	Lack of commitment from the management	*This country! I can’t say if we will ever achieve it. If it is private hospital, yes. But for Primary Health Centre that is owned by government it is not achievable*	This transformation of quality service is yet to be fully demonstrated in our PHC setting. The national interest to enhance the quality of health care service delivery to the people is poorly addressed
	Low empowerment	*I am a community health nurse who had first degree in nursing and knows the importance of community centered care…the people will respond back based on what you do, whether it is quality care or poor care. But the patients are too much for the staff. So, I think it might be a bit difficult…*

**Table 2:** Themes, sub-themes, and codes with meaning based on positive perceptions

Themes	Sub-themes	Codes	Meaning
Outcome driven healthcare	Being compassionate and caring	*I think it is a positive concept with good strategy which should be implemented…It will make health care delivery appropriate for the client, so I think it is a good concept* *It is very good if we can integrate it in the service, especially at the primary health care centre*	Nurses revealed committed as healthcare provider to patients to give holistic healthcare service that is geared towards helping the community member to contribute to their own health care and collaborate
	Enrich nursing care	*It will give a better outcome in the care that is been given to the clients* and *enhance the practice of nursing*	
Valuable care provider	Increase information sharing	*…This will be great for the nurses and the community because both of them will benefit from it*	Participants reported increased advantage by quality of care for patients through deliberate changes in caring for patients to bring better health outcomes
Driven healthcare service	Compel to do normal thing	*It will be reminding us that these are the things we need to do…So, I think it will be very helpful, it will be very helpful and it will tailor the way we interact with our patient*	The importance of PCC knowledge was reported by the participants to be able to interact well with patients. Although the concept is good the majority of them are unaware of it
	Acceptance of usefulness	*PCC is necessary because taking care of a patient or a client, especially in a primary health care centre, it will be good to create more awareness to among people generally…*	
Communication to sharpen care	Understanding information	*To me, I think PCC in the PHC is relevant because it will improve the quality of care when you allow clients to participate, to talk to you freely and if you listen to her, you know we will work together because they will see us as friendly*	Effective communication was identified by nurses as a means to improve healthcare. Participants acknowledged the importance of listening to patients’ expression and to understand them as they pour out their emotions and anger during care
	Interpersonal relationship	*You need to make the people feel important because I am also a human being, you know, when we treat patients with respect. I think we need to improve on that*

**Table 3:** Demographic characteristics of the participants and perceptions

Participants code	Gender	Age	Year of working experience	Educational level	Location of PHC
P01	F	31	02	RN, BNSc	Ede-North
P02	F	30	10	RN, BNSc	Ede-North
P03	F	37	11	RN, BNSc	Ife-South
P04	M	33	10	RN, MSc	Ede-North
P05	F	37	11	RN, BNSc	Ife-Central
P06	F	35	10	RN, BNSc	Ifetedo
P07	F	40	11	RN, RM	Oshogbo
P08	F	30	6	RN, BNSc	Olorunda
P09	F	38	10	RN, BNSc	Ife-Central
P10	F	34	06	RN, BNSc	Ifelodun
P11	M	30	11	RN, BNSc	Egbedore
P12	M	33	12	RN, RPHN	Ejibo
P13	F	40	16	RN, BNSc	Ede-North
P14	F	40	15	RN, RM	Ife-Central
P15	M	42	17	RN, BNSc	Ife-Central
P16	M	35	15	RN, BNSc	Irepodun
P17	M	40	12	RN, RM and RPHNN	Ife-Central
P18	F	38	15	RN, RM	Ejibo
P19	F	39	16	RN, BNSc	Egbedore
P20	F	33	12	RN, RM	Ife-Central
P21	F	39	15	RN, RM	Olorunda
P22	F	41	15	RN, BNSc	Ife-South
P23	F	30	12	RN, RM	Ifetedo
P24	F	38	15	RN, BNSc	Ifelodun
P25	F	45	18	RN, BNSc	Ede-South
P26	M	41	15	RN, RM and RPHNN	Ede-North
P27	F	50	18	RN, RM and RPHNN	Oshogbo
P28	F	39	15	RN, BNSc	Ife-East
P29	F	35	12	RN, RM	Irewole
P30	F	42	18	RN, RM and RPHNN	Ede-North
P31	F	41	12	RN, RM and RPHNN	Ife-Central
P32	F	48	26	RN, RM and RPHNN	Ifelodun
P33	F	51	25	RN, RM and RPHNN	Ede-North
P34	F	59	31	RN, RM and RPHNN	Ejibo
P35	F	48	19	RN, BNSc	Ede-North

The theme poor approach by the nurses comprise of two sub-themes based on the participants’ narrative: (I) sentimentalism of the nurses (ii) uncaring approach.

### Sentimentalism of the nurses

In this study, majority of the participants reported that although nurses play an important role in the healthcare system with regard to patient care. However, delivery of holistic care to patient requires a lot of time and consume time which the nurses don’t have due to the large in flow of patient. The expression of the participants is the experience of nurses in the PHC with the inflow of patients. This means that increase in population of patient compared with the manpower available may be a vast challenge to PCC. Below revealed the evidences:

*“…I think it might be a bit difficult because the patients are too much for the staff. I am a community health nurse who had first degree in nursing and knows the importance of a community centered care…the people will respond back base on what you do, whether is a quality care or a poor care P35”.*

“To treat patient holistically it consumes time and at time to some patient they will think is it not relevant P19”.

*“Not all nurses can use it and because at times the patient might be too much for the staff… so is not practicable, the patient centered care is not practicable in Nigeria presently because most of PHC centre in Nigeria has turn to something else P13”.*

### Uncaring approach

The participants from this study presented a narrative of uncaring approach of the nurses towards patient as one of the hurdle to effective use of PCC among health professionals in the PHC setting. Flawed PCC approach was reported by the participant due to observed tensions in the interaction with the patients and families. It is hope that an improvement in the compassionate and empathy from nurses will increase the utilisation of PCC approach. This is well exemplified by the excerpts interview:

*“Instead of coming to health centre they go to the traditional healer setting because they believe that they are religious that they care about their religion but if we now bring the approach into healthcare setting it will increase their flow P33”.*

*“Some patient when they come to us, to confined in us but when they tell us their secret immediately they left we will just be sharing their secret with others which is not supposed to be… P17”*

Lack of enforcement agency: Two sub-themes emerged from the theme lack of enforcement agency: (i) lack of commitment from the management (ii) low level of empowerment.

### Lack of commitment from the management

Participants’ narrative during the interviews reported that there is poor organisational commitment to obligation towards ensuring quality healthcare services to the people in the rural communities. It was mentioned that PHC leadership commitment to quality healthcare will determine how far nurses can work with this concept in spite of the evidence-based and scientific studies on PCC. The nurses also expressed that the management has given no attention to PCC concept when we consider the way in which PHC are operating today in Nigeria. Therefore, there is need for government through other stakeholders to enable the nurses use PCC by providing good infrastructure, conducive consultation rooms, and human resources. The quotes below exemplified it:

*“If there is no enough staff it becomes a challenge, you understand…people centered care is a very good concept but in Nigeria the government part is there, the community part is there. When we put patient centered care into the healthcare and government failed to do their part to favor this new care, that’s my own problem P27”.*

*“If we look at the technicality aspect of it, we will see that Nigeria PHC is still far back in the sense that we might not be able to do it optimally…For example, look at our infrastructure, we don’t even have a comfortable chair to sit when we come to duty. I think the government needs to step up first… P31”.*

### Low level of empowerment

Participants reported the importance of resources such as: equipment, training, personnel, skills upgrade of nurses, suitable infrastructure as enabling factors and motivation to PCC action. When these enabling factors are inadequate in the PHC environment, the nurses cannot do much. Therefore, poor demonstrations of PCC at the point of care was attributed to weak enabling factors from the government and organisational level of the health system. The role of the government through Nursing Council body and other agent thus play an important function to successfully implement PCC at the grass root level of the healthcare system. Otherwise, quality healthcare service to the community will continue to be jeopardized. The extracts below are evidence to support:

*“…. There is low empowerment in this service, there is different things like that… Normally before when we still have good times in Nigeria we do have seminars most of the time then to upgrade our skills but now there is no such things again... P34”*

*“How can we practice patient centeredness when some PHC centers don’t even have any nurses? In some local government, we have only one qualify nurse in the whole local government area, how are we going to manage that clinic and still give patient quality care? We will not be able to do it and that is why the government need to employ more staff P19”*

Outcome driven healthcare theme emerged with two sub-themes: (i) Being compassionate and caring and (ii) Enrich nursing.

### Being compassionate and caring

This sub-theme being compassionate and caring emerged in this study as participants described their experience on PCC concept that could help people get better quality healthcare service. The ability of the healthcare professionals to express caring and deliver healthcare service with compassion, knowledge and skills make nurses not just compassionate but leads to competent nursing actions. Nurses expressed awareness of quality nursing practice includes respect and value of patient, preference of patient care. The following quotes aptly support this:

*“PCC is a positive concept if we have the empowerment… and it will be significant for the nurses and the community because both will benefit from this concept. Yes, I think PCC is necessary especially in primary health care center, because taking care of patient or a client is not about the patient having a particular disease but your ability to empathize with the patient and give adequate supportive nursing intervention…P05”.*

*“You made people feel very important when you treat your patient with respect… patients will be able to trust in us and open up to you, also report on time if they have any problem” P07*

### Enrich nursing

It was revealed that providing nursing care with PCC concept will enrich nursing practice. Participants reported that nurses will have opportunity to actively involve patients to take action through participation, involvement and collaborative care towards the needs of patient. Participants mentioned that nurses are known to be agent of change through their actions in time past. However, PCC could play a key role in nursing practicing, and improve the quality of care to change patient situation. The extracts below speak volume of this:

*“The first place of contact with the patient is the primary health care so I think is good to introduce client centered care to enrich the practice of nursing care to our patient P11”.*

*“There are many benefits in using patient centered care for the community. One of it is that it can decrease the rate of maternal and child deaths when the people are given quality nursing care and they are satisfied with the way a nurse treat them P03”.*

*“…if you respect your patients, all that they cannot tell you before, they will begin to tell you and this will enhance the quality of care nurse give because now you can understand what the patient is going through aside the sickness P20”*

The theme valuable care provider emerged with one sub-theme: increase information sharing.

### Increase information sharing

In this study, participants reported using PCC approach to care for individuals will increase information sharing. Thus, enhance and increase patient nursing care practices, improve nurses’ performance when patient have sufficient knowledge that can promote good health and contribute to better nurse-patient relationship. The extracts below bear eloquent testimony to this:

*“If PCC concept is been encouraged at the PHC level the status of the patients’ health will improve significantly PHC health system will also improve because it will promote quality care…P08”.*

“To me is an interesting way of caring for patient because it gets to increase their knowledge and make your job easier…P01”.

Driven Healthcare service: This theme emerged with two sub-themes: (i) Compel to do normal thing and (ii) Acceptance of usefulness.

### Compel to do normal thing

Participants revealed that PCC concept is good because it gives room for ideal nursing care practice in the PHC. Furthermore, it was reported that government need to support PCC concept and provide all facilities to enable nurses and other healthcare providers implement the strategies of PCC during healthcare delivery service. The extracts below speak volume of this:

*“PCC approaches is a useful concept that will remind us of the manner we should address our patients. So I think it aid nurses to work well with their client if all the necessary resources are made supply and if government bureaucracies permit it...P21”.*

“I think is a good concept with good strategies that should be integrated in our nursing health care delivery practice because is appropriate for client…P10”.

### Acceptance of usefulness

It was reported by the participants from this study that nurses’ role in delivery quality healthcare service in line with PCC strategies involve time with the patient and comfort. They expressed that although this type of experience with patient will improve patients feeling because it respects individual value and cultural beliefs. Also it will bring agreement of action between the nurses and the people. Therefore, PCC policies modifies care by not focusing on the disease process alone but treat the person holistically. So, is useful in caring for patients. These extracts below are evidence:

“It would enhance the practice of nursing if we can incorporate PCC in our nursing care at the grass root level, it will go a long way to impact the community P04”.

“As far as nurses and nursing is concern we support PCC but I think there is need to improve on mode of healthcare delivery service P35”.

Communication to sharpen care: This theme has two sub-themes that emerged: (i) Understanding information and (ii) Interpersonal relationship.

### Understanding information

The narrative by the participants revealed that nursing professionals need to improve on how sharing information with patient at the level the people could comprehend and understand what needs to be done effective and efficient health outcome to occur. The healthcare professionals must give amply opportunities for the people to be well informed and engaged in their own healthcare management. The below comments exemplified this:

“It will improve the service we give to our client and likewise effectively contribute to their health…P16”.

*“The PHC health system and PCC practice is important for improvement on the quality of care. You know we work at the grass root where we see different types of patients from poor economic background, so it important you allow your patients to participate in whatever care you are planning for them P25”.*

### Interpersonal relationship

Interpersonal relationship emerged from this study as participants reported on the benefits of PCC concept to quality, healthcare with the patient when there is good communication between nurses and the patient. It was further expressed that although nursing profession support the use of PCC for quality healthcare improvement and agreed with the concept, however, there is need to upgrade their practice. This will help the nurses to give a more individualised healthcare with good understanding. The below extracts reflect this:

“…it allows patient to have access to quality healthcare delivery because they will see the nurses as friends and relate with us freely P21

“I think the nurses should learn more about this profession to be able to practice nursing more effectively P31”.

*RN* Registered nurse; *RM*: Registered Midwife; *BNSc*: Bachelor of nursing science; *RPHNN*: Registered Public health nurse; *MSc*: Masters of nursing science

## Discussion

The core element of evidence-based care practice in the health system is patient’s value and preferences which are part of the principles of PCC concept to empowered patients to take active responsibility in the sharing decision of their health care [66]. This study acknowledges the importance of delivery PCC and the involved positive impact associated with the patient receiving care and the nurse delivering the healthcare service. However, the provision of PCC in nursing healthcare remain a challenge in the PHC setting. A study by Moore et al. [67] reported on barriers facing PCC implementation which is identical to our findings. Our findings revealed that PCC is of significant importance to healthcare professionals, institutions and other stakeholders in order to achieve physical, social and mental wellbeing of the people in the primary health care setting in Nigeria. There is need for improved performance of nursing care and the quality of healthcare services delivery to the people in the PHC health system.

Although our study identifies uncaring approach and nurses’ sentiments as one of the hurdle to proper utilization of PCC, the knowledge and practice of PCC in the PHC will not only enhance the quality of care but also reduce patients cost especially in primary health care system of Nigeria where patients pay for everything such as disposable glove, needle and syringe just to mention few. This is because in Nigeria, healthcare delivery services with PCC has been exaggerated. Davison and Williams [68] argued a different findings that the problem with healthcare professionals is how caring is viewed and defined in their personal definition instead of fitting into the global standard. This resulting in nurses having little view about patient. Kuipers et al. [6] also argued that most of the healthcare service at the grass root health system are not patient-oriented despite the scientific evidence of patient satisfaction with PCC [6].

The central mission of healthcare is PCC. Nurses considered time as a challenge to the delivery of quality healthcare using the PCC approach due to their sentimentalism since it required a considerable amount of time in practice. For instance, this recording confirms this complaint from one of the nurses interviewed: “*We look at the time for our patient…we don’t have enough time to listen to the patients to say what they want to say.”* This results in poor interpersonal relationship and communication. A study by [68] reported similar challenge and stated that the reason why nurses have problem with time is because they give priority to daily routines instead of focusing on holistic caring of the patient.

Whereas, the foundation of better quality healthcare service to the patient is good communication and positive interpersonal relationships between nurses and patients. Communication plays an important role in holistic patient healthcare services and this responsibility falls on the nurses [69]. It is essential for the patients to understand the information provided by the nurses. Such a strategy improves nurses’ performance, increases active patients’ input on health matters, reduces misunderstanding between nurses and patients, increases awareness of preventive healthcare and improves healthcare outcomes. Misconceptions and negative perceptions hinder the nurses from developing effective nurse-patient relationships [69, 70]. The implication of this is that it affects the mission and objective of healthcare which is awareness of the causes of disease for prevention and promotion of quality healthcare, and rehabilitation [71]. In addition, the universal health coverage for all by the 2030 according to WHO may never be achieve. if promptly intervention is not given.

It was observed that the PHC health system was subject to political influence and management in Nigeria. Additionally, the political influence and socio-economic factors surrounding the PHC system in South-West Nigeria also influenced the nurses’ negative perceptions on implementation and practice of PCC in the PHC centres. According to WHO, effective use of PCC in the PHC program has the potential to contribute to patients assess to quality healthcare with improved Health outcome [72]. It is therefore, recommended that the PHC centres and the surrounding factors be improved in order to achieve these goals. Lack of understanding and shared view between lawmakers and the healthcare professionals impacts healthcare practice and the health system [66]. The government has depraved many of basic human right items of right to access quality healthcare [22].

The existing disparity in remuneration among nurses working in the local government compared to the state and federal hospital nurses was significant according participants’ narratives during interview. This could have also affected the nurses’ perceptions of PCC negatively. A study by [73] is line with our findings. It reported that nurses are dissatisfied with their job when they are poorly remunerated because it affects their well-being and personal life. We therefore, recommend that nurses should be well remunerated in the interest of the people of the community. However, PCC are still lacking when relating to patient and the effect is when care is not tailored towards patient [6].

Even though in nursing profession the concept of PCC is comprehensive and valued [10], yet our study revealed that the principles are lacking in practice among the nurses working in the PHC. As a result, the patients are not receiving quality healthcare delivery service. This authors Kuipers et al. [6] share the same opinion in their study.

The majority of the nurses who expressed full support to the implementation of PCC in the PHC was observed to have a Bachelor degree in Nursing education and were aged between 30—48 years with quite a handful holding a diploma in nursing. This can be attributed to the fact that nursing education at these levels covered theoretical concepts on PCC, thus providing these nurses with the necessary knowledge and PCC expectations. However, the lack of emphasis on PCC education in nurse training institutions generally was observed from this study. These findings are similar to the results presented in other settings by these authors [35, 67, 74].

A significant number of the nurses interviewed indicated using the PCC approach in their nursing practice. However, this was not observed at all at the various PHC settings under study. This was attributed to nurses’ misconceptions and personal view about PCC concepts and practices [68]. Nkrumah and Abekah-Nkrumah, [75] study mentioned that nurses are faced with some barriers in using the PCC approach which could leads to the lack of expertise among them for PCC practice. We belief that this fact may have contributed to the negative perceptions recorded from our findings.

There is need to ensure that all graduates and registered nurses are well informed and knowledgeable in the theories and practice of PCC in nursing care. This would help in achieving quality and positive healthcare outcomes. Additionally, the patient would be treated with respect, dignity, value, compassion and would have a sense of satisfaction from the service provided to them. Such training of nurses with PCC would also enable and empower them with the necessary knowledge and skills for improved nursing profession in any healthcare system. Our findings was found to be similar to those presented by these authors [76, 77]. Nursing education on PCC is one of the ways to improve the nurses’ experience using PCC. It is also worth mentioned that [78] stated that nursing care quality in terms of the evidence show the strength of nursing as a noble profession has greatly declined in many countries, and so, PCC is not obligated [78].

Nurses have the potential and ability to influence and contribute to improved quality healthcare services and nursing practice in Nigeria need to fully embrace PCC as a means of healthcare service delivery. The poor demonstration of PCC reported in PHC can be attributed to existing knowledge gaps among nurses, lack of enforcement from the stakeholders and inadequate training to upgrade nurses’ skills and knowledge, among others. Appelgren, Bahtsevani, Persson, and Borglin [79], reported similar issue as presented in our findings that RN have negative attitudes, poor delivery of healthcare service to patient due to their perception, while [80] submitted that nurses no long meet the needs of their patients. Therefore, nurses with diploma education would benefit greatly from a postgraduate education that has PCC nursing model. Thus, reduce inequalities of care.

In addition, valued care providers required increased information sharing between the healthcare providers and the patients. Hence, promoting the participation, collaboration, and involvement of patients in the decision making process of their healthcare, would inevitably improve cooperation from patients to enable nurses understand the patients’ perspectives, and explore patients’ concerns and ideas, as well as their experiences regarding their illnesses [72].

The cooperation between nurses and patients through interaction with PCC would drive positive outcomes in nursing care, improve performance, enhance community experiences of healthcare services, and increase patients’ satisfaction [81]. Thus, promoting high quality healthcare services through effective community involvement is ensured when PCC is well integrated in the PHC system. The patients will be provided with full support and assistance to understand what is required of them when accurate and clear information is provided through effective partnership and collaboration. This will shift care from the traditional authoritarian nursing care to patient-centeredness and the people benefit at the same time, promoting wellness.

## Conclusion

There is need for continuous training, and upgrading of nurses in line with global recommended standards of providing quality healthcare service delivery to the people. Therefore, the federal and state governments and local government council through the Nursing Council body should regulate, supervise, monitor and enforce the use of PCC in the PHC healthcare system. It is also recommended that government should provide all the necessary resources and support to nurses and other healthcare providers in the PHC to ensure quality care services to the community members by creating an enabling environment for nurses and other health care providers to facilitate the provision of PCC in the PHC.

## Data Availability

The data analyzed during this study that supporting our findings are available in the manuscript as supplementary.

## Instrument for the individual interviews guide

**Table:**

Pseudonym/identification code	
Code for PHC centre	

## Introduction

### Good morning/day! Thank you for agreeing to meet with me and share your views.

As you may know, the purpose of this interview is to help us understand the practices of patient-centered care and your experiences.

Kindly let me review some important considerations before we begin the interview. I am recording this interview to ease the analysis of this qualitative data. However, all responses will be kept highly confidential.

By signing the consent form, you confirm that you have consented to participate in this study and that the interview discussion can be audio recorded.

The researcher is interested in both negative and positive comments and often the more challenging and in-depth comments will be most helpful.

### Icebreaking

Can you kindly give me a short profile of yourself (Current position, how long since you have joined the PHC, and your academic qualification and your age)?

### Main interview questions

1. No doubt, the nursing profession has come a long way in Nigeria. From your experience, what has been the mode(s) of delivery of nursing healthcare services in PHC in Nigeria? Have there been changes? If yes, would you like to share with me some of these changes?
2. Could you please describe your understanding of the concept of patient-centered care? Are you using Patient-centered care as part of your healthcare delivery strategy? Please kindly elaborate: probing questions (Approach)
3. What is your perception on PCC in terms of what it is, quality of care, relevance, strengths, weaknesses, and suitability for improving quality nursing healthcare services in Nigeria PHC?
4. Which of the strategies do you commonly use at the point of care with patients during healthcare service? Probe: why?
5. Probing: Do you have any suggestions or solutions to the situation described?
6. To what extent are nurses ready to implement the PCC approach solutions?
7. Are there contextual factors necessitating the adoption of PCC in PHC in Nigeria? If yes, can you shed more light on these factors?
8. What factor(s) do you foresee as hostile to the implementation and use of PCC for the nurses?
9. If we are to embark on developing an implementation model as a guide for nurses in PHC in Nigeria, what specific suggestion do you have, in terms of design, content, and implementation?

Thank you for your contributions.

## Abbreviations

PCC: Patient-centered care
PHC: Primary health care
WHO: The World Health Organization
LGAs: Local government areas
LMIC: Low and middle-income countries
RN: Registered nurse
RM: Registered Midwife
BNSc: Bachelor of nursing science
RPHNN: Registered Public health nurse
MSc: Masters of nursing science
